# Graphene-Enabled Electrodes for Electrocardiogram Monitoring

**DOI:** 10.3390/nano6090156

**Published:** 2016-08-23

**Authors:** Numan Celik, Nadarajah Manivannan, Andrew Strudwick, Wamadeva Balachandran

**Affiliations:** 1 Department of Electronics and Computer Engineering, Brunel University London, Kingston Lane, Uxbridge UB8 3PH, UK; nadarajah.manivannan@brunel.ac.uk (N.M.); wamadeva.balachandran@brunel.ac.uk (W.B.); 2 2-Dtech, Core Technology Facility, Incubator Building, 46 Grafton Street, Manchester M13 9NT, UK; andrew.strudwick@2-dtech.com

**Keywords:** graphene, electrocardiogram (ECG), long-term monitoring, dry electrodes, contact impedance, Raman spectroscopy

## Abstract

The unique parameters of graphene (GN)—notably its considerable electron mobility, high surface area, and electrical conductivity—are bringing extensive attention into the wearable technologies. This work presents a novel graphene-based electrode for acquisition of electrocardiogram (ECG). The proposed electrode was fabricated by coating GN on top of a metallic layer of a Ag/AgCl electrode using a chemical vapour deposition (CVD) technique. To investigate the performance of the fabricated GN-based electrode, two types of electrodes were fabricated with different sizes to conduct the signal qualities and the skin-electrode contact impedance measurements. Performances of the GN-enabled electrodes were compared to the conventional Ag/AgCl electrodes in terms of ECG signal quality, skin–electrode contact impedance, signal-to-noise ratio (SNR), and response time. Experimental results showed the proposed GN-based electrodes produced better ECG signals, higher SNR (improved by 8%), and lower contact impedance (improved by 78%) values than conventional ECG electrodes.

## 1. Introduction

Cardiovascular diseases are the leading cause of death worldwide, killing more than 15 million people every year [1]. Continuous and real-time monitoring of the heart activities with well-established medical test electrocardiography (ECG) plays a very important role in early diagnosis and treatment of cardiovascular diseases. The ECG waveform that reflects the electrical activity of the heart is widely used for early detection and management of heart problems. Traditionally, the most commonly used bioelectrodes are of the gel-type disposable Ag/AgCl electrodes [2] and these are simple, reliable, and cost effective during cardiac monitoring. These standard wet-type electrodes typically have three main parts: Ag/AgCl layer for sensing; conductive gel to maintain good electrical contact with the skin; and a connector part for conducting the electrical signal through third-party devices for monitoring purposes. On the other hand, it is not suggested to use these wet electrodes in long-term cardiac monitoring due to skin irritation and allergic reactions. Studies have showed that using adhesive gel on wet Ag/AgCl electrodes can trigger dermal irritation and cause potential signal degradation in long-term measurements [3]. Therefore, the demand for comfortable and easy-to-use electrodes has resulted in fabrication of dry or gel-less ECG electrodes that eliminate the need for gel and even skin preparation.

Several attempts have been made in fabrication of dry-contact ECG electrodes made of metallic and silicon substrates that penetrate the skin surface for electrophysiological measurements [4,5,6]. Although this method is clinically invasive and gel-less contact with the skin, it is difficult to satisfy electrical stability between skin and the electrode. Therefore, the applications with dry-contact ECG electrodes need to be investigated further for long-term measurements. Another promising approach is based on capacitive coupling using non-contact type dry ECG electrodes [7,8]. These types of electrodes are classified as a non-contact because the electrodes are isolated with an insulating layer from the skin with hair, clothing, air, or other synthetic or textile fabrics. In non-contact electrodes, the risk of skin irritation and preparation is eliminated; the deployment of the electrodes to the skin is easy and safe, and integration with other conductive fabrics or textile is possible. However, they have issues with contact impedance and motion artifacts throughout capacitive-coupling ECG signal from the body [9]. To overcome these limitations of dry electrodes, researchers have investigated the use of nanomaterial-based electrodes in long-term ECG sensing due to their properties of high electrical conductivity and flexibility for better wearable electronics. Myers et al. [10] developed a silver nanowire-based (AgNW) dry electrode for ECG monitoring where the NWs were integrated below the surface of the polydimethylsiloxane (PDMS) layer on the dry electrode. The electrode was used on a wristband with a conductivity of ~5 × 10^5^ S/m at 50% tensile strain. The skin–electrode contact impedance was decreased by applying a mild pressure onto the AgNW electrode, and the results demonstrated that the AgNW/PDMS dry electrode outperformed the pregelled Ag/AgCl electrodes with fewer motion artifacts when the subject was swinging their arms and jogging. Due to interesting mechanical and electrical properties of carbon-based nanomaterials, carbon nanotubes (CNTs) are selected to disperse into the flexible polymer substrates to make a better conductive ECG electrode. Lee and his colleagues [11] fabricated a dry ECG electrode by dispersion of CNT into the PDMS layer to investigate the effects of CNT concentration and robustness to motion and sweat. Tests showed that there was no sign of signal degradation after continuously wearing the CNT/PDMS electrodes after exercise (in the presence of sweat), and for 7 days. In addition, the results revealed that there was no skin irritation after wearing the electrodes on the forearms for 7 days. However, the main point for developing flexible electrodes lies in flexible substrate and conductive material. Conventional methods such as gel, contact, and non-contact dry-type of ECG electrodes tend to have low charge carrier density, thus causing low conductivity and lower signal quality. Although nanomaterials-based electrodes provide a better signal-to-noise ratio (SNR) with flexibility, and eliminate skin irritation problems, there are still shortcomings to be resolved in long-term ECG monitoring for better accuracy. Previous studies indicated that metals deposited on PDMS exhibited wrinkles and cracks frequently [12,13]. Additionally, it is difficult to achieve a proper mixing process due to the stickiness of polymer. Besides, an experiment showed that the adhesion between metallic layers and PDMS substrate is poor [14]. More importantly, the conductivity of the conductive polymer is not as good as a metal conductor (80 S/m versus 5 × 10^5^ S/m) [5]. For further development of conductive and flexible electrodes into wearables, it is important to use highly conductive (~10^7^ S/m) and thin metals in such a reliable, stable, and long-term ECG sensing application.

Graphene (GN), a single-layer two-dimensional structure nanomaterial, exhibits exceptional physical, electrical, and chemical properties that lead to many applications from electronics to biomedicine. The unique parameters of GN—which has notably the highest electron mobility (~200,000 cm^2^·V^−1^·s^−1^), the highest thermal conductivity (5300 W·m^−1^·K^−1^), the highest surface area to volume ratio (2630 m^2^/g), the fastest moving electrons (~10^6^ m/s), and is the best conductor of electricity (resistivity of 10^−6^ Ω·cm) in any material—are bringing heightened attention into biomedical applications [15]. In this study, we fabricated a dry electrode by growing GN within copper (Cu) substrate on top of a silver (Ag) layer of a commercial ECG electrode using a chemical vapour deposition (CVD) method. Based on this technique, we produced a GN-based ECG electrode for the first time on the development of ECG electrodes with GN substrates. We measured contact impedance according to frequency changes and compared the results with those of conventional Ag/AgCl electrodes. Furthermore, we measured ECG signals and quantitatively compared the performance of the new GN-based ECG electrodes against wet electrodes. To determine the feasibility of long-term monitoring, we studied the influence of GN-based electrodes on 10 people. We tested these electrodes for one person in each week and demonstrated their robustness in ECG monitoring.

## 2. Materials and Methods

### 2.1. Fabrication Process

**Figure 1:**
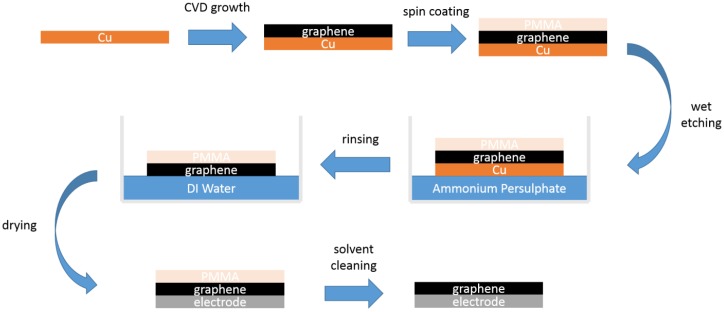
Schematic diagram showing coating process for synthesizing graphene-coated electrode.

A number of different algorithms for synthesizing GN have been reported since first obtained in 2004 by Novoselov and Geim, including mechanical exfoliation, liquid-based exfoliation, epitaxial growth on silicon carbide, and chemical vapour deposition (CVD) growth GN on metal substrates such as silicon, nickel, and copper [15]. To fabricate a GN-enabled conductive electrode, we have used the CVD approach (Figure 1) for coating GN on top of a metallic substrate of the commercial ECG electrode. This has advantages on transition into different metals such as Ni, Cu, and Si substrates, and CVD-grown GN is inexpensive and a feasible method for single-layer GN synthesis compared to other production techniques.

We coated GN as a layer onto the target electrodes after GN growth on a metallic substrate and transfer processes, which were carried out by a specialist graphene company [16]. The GN was produced utilising hydrogen (H_2_) and methane (CH_4_) gases based on the chemical vapour deposition (CVD) growth procedure, similar to those used elsewhere in the literature on copper (Cu) substrates [17,18,19] to yield monolayer graphene films. These films were subsequently transferred to a Ag layer of the target electrodes via a wet chemical approach to etch away the Cu substrate, involving a spin-coated Polymethyl methacrylate (PMMA) support layer which is later removed from the GN–Ag substrate form with solvents, akin to work carried out elsewhere [20,21]. The details of growth and transfer processes can be seen in Figure 1, which shows the fabricated electrode within a very thin GN layer—which was about 3.7 Å (0.37 nm)—for ECG sensing applications. Electrical properties of the GN-coated electrodes were measured at room temperature with a two-probe method using a digital multimeter (0.1 Ω–2.8 Ω). The electrical conductivity (σ) of the electrode before and after GN coating was measured as 2.48 × 10^2^ S/m and 6.94 × 10^4^ S/m, respectively.

### 2.2. Raman Spectroscopy and SEM Images

GN coating process can be repeatable on a variety of common metallic crystals such as copper (Cu), nickel (Ni), silicon dioxide (SiO_2_), platinum (Pt), gold (Au), and silver (Ag). Cu is ideal metallic crystal for GN growth because of its low carbon solubility, and the carbon dissolution at high temperature into the bulk helped in self-termination of monolayer growth [22]. Furthermore, Cu is selected here for fabrication of the ECG electrodes due to the high deposition of GN while transferring GN onto the Ag substrates of the electrodes. Raman spectroscopy and scanning electron microscopy (SEM) analyses were done in order to confirm the presence of the coating of GN on the fabricated electrode after completing the transferring process. SEM analysis was done by the Zeiss Supra field emission electron gun (FEG)-SEM and Raman spectroscopy analysis was done using a confocal Raman spectrometer that was available at 2-D Tech [16].

**Figure 2:**
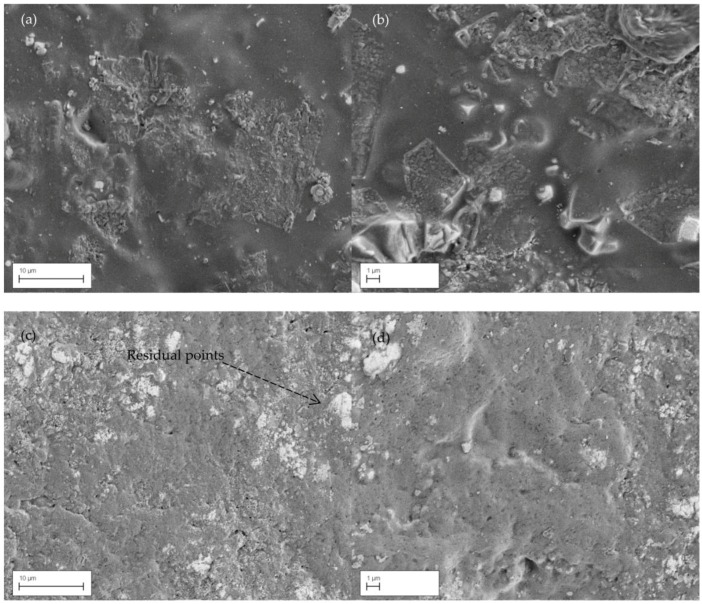
Scanning electron micrographs (SEMs) showing: (**a**,**b**) SEM of dry electrode before coating; (**c**,**d**) graphene-coated dry electrode.

As can be seen from Figure 2, GN has expanded through almost the entire cross-section of the electrode, and which was designed to increase the contact area between the electrode and skin. These porous structure layers were observed under the SEM before and after GN coating process. SEM images of GN-coated electrode were taken after several experimental studies. Because of this, patterns of residual points were detected just above the GN layer on the surface of the electrode (as white colour in the Figure 2). These images further show that GN coating has smoothed the surface and hence can improve the skin-to-electrode contact and detect better ECG signals.

**Figure 3:**
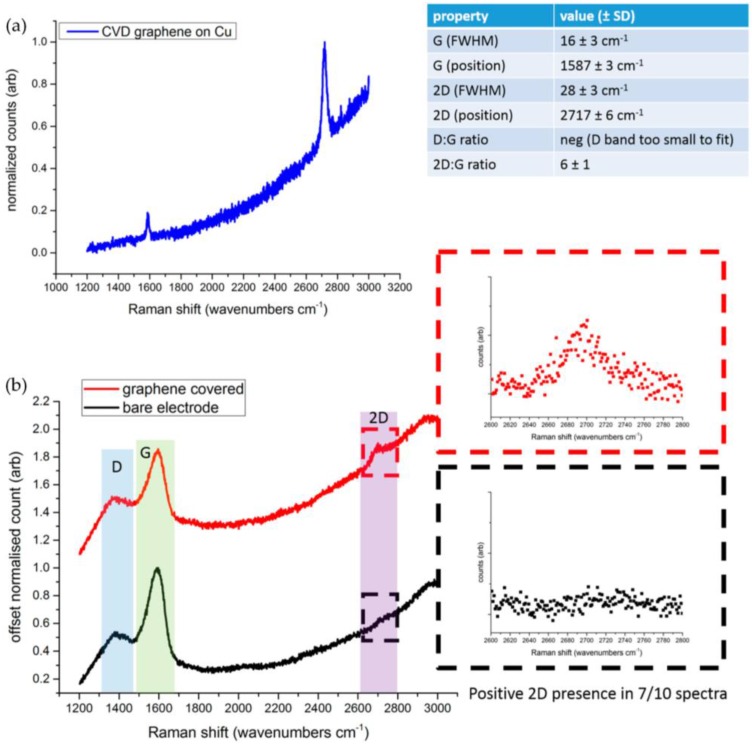
Raman analysis of graphene (GN) and bare Ag/AgCl electrode. (**a**) Raman spectrum of graphene grown on Cu after chemical vapour deposition (CVD); and (**b**) Raman spectrum for the GN-coated electrode and conventional Ag/AgCl bare electrode.

Optical detection relying on light scattering is especially attractive because it is fast, sensitive, and nondestructive. In particular, Raman spectroscopy analysis is the most powerful technique available for the characterization of carbon nanostructures such as nanotubes and graphene [23]. The Raman spectrum of carbon-based materials shows two main peaks, the G- and D-peaks, which lie at around 1580 cm^−1^ and 1360 cm^−1^, respectively (Figure 3b). The G-peak corresponds to the E_2g_ phonon at the Brillouin zone center. The D-peak is due to the breathing modes of sp^2^ atoms and requires a defect for its activation [24]. However, the most prominent feature in graphene is the second order of the D-peak: the 2D-peak. This lies at around 2700 cm^−1^ (Figure 3b) and it is always seen, even when no D-peak is present, since no defects are required for the activation of second-order phonons. A supplementary material file has been added to this work as it shows the Raman analysis of bare Ag/AgCl electrode and Graphene-coated electrode respectively in Figure S1, and Figure S2 in-detail for detecting 2D band in 10 measurements. Its shape and position distinguishes single-layer from multilayer samples. Regarding our tests, the Raman spectra of GN and GN-coated sample was measured by taking 10 point measurements across the whole sample surface at 488 nm excitation. In an attempt to get clearer data and rule out any fluorescence signals from the underlying substrate, 10 data points were subsequently obtained using a 514 nm laser. Figure 3 shows the Raman spectrum of GN grown on Cu (Figure 3a) and for both GN-coated electrode and bare Ag/AgCl electrode (Figure 3b) obtained from the 488 nm laser. As can be seen from the images, three important bands (G, D, 2D) were observed in Raman spectrum and separately 2D-peaks were selected to be analysed.

According to Figure 3a, Raman spectra was measured for the single-layer graphene (GN) area for the sample which was grown on Cu after CVD. A shift in the 2D-peak position of single-layer GN has been observed in the range of 2711–2723 cm^−1^. The value of full width at half maximum varies in the range of 25–31 cm^−1^ which represents the single layer of GN [25]. The ratio of 2D/G also indicates that the sample was grown by single-layer GN, and this ratio for this sample has been varied in the range of 5–7. Figure 3b shows the comparison of Raman spectra for the GN-coated electrode and conventional bare Ag/AgCl electrode. As can be seen from this figure, the presence of the 2D-peak in the GN-coated electrode (in red square) differentiates the presence of GN from the bare electrode.

### 2.3. Measurements of ECG Acquisition System

**Figure 4:**
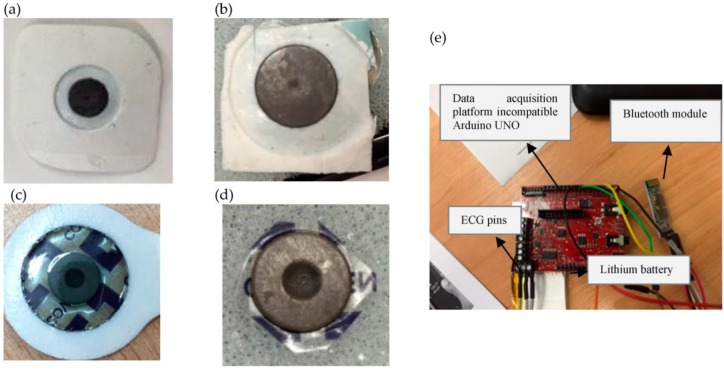
Experimentally used electrodes and data acquisition system. Bigger size of electrode (Ambu) before GN coating in (**a**), and after GN coating in (**b**); smaller size of electrode (Covidien) before GN coating in (**c**), and after GN coating in (**d**); data acquisition unit in (**e**).

ECG signals were separately measured from proposed GN-based electrodes and conventional pre-gelled Ag/AgCl electrodes (Ambu) via ECG acquisition system (e-Health and Arduino) using 3-leads to allow measurements from multiple biopotential electrodes. Figure 4 shows both types of electrodes (GN-based and conventional pre-gelled ECG electrodes) used in the experiments. In order to obtain the electrocardiogram, ECG lead-1 configuration was conducted throughout experimental testing of ECG electrodes on a 29 year old male; two active electrodes were attached to the left and right chest and the other driven-right-leg (DRL) electrode was attached onto the left waist for referencing. ECG signals were filtered between 0.5 and 100 Hz and an additional notch filter was applied at 50 Hz cutoff frequency during sampling and amplifying of ECG signals at the signal acquisition system, which is shown in Figure 4. We have coated GN on two types of electrodes which were (1) slightly bigger Ambu type of electrode and (2) smaller size Covidien type of electrode. Figure 4a,b shows the Ambu type of electrode just before and after GN coating, respectively. This type of electrode has a diameter of 38 mm and a thickness of 1 mm. Likewise, the Covidien type of electrode is shown in Figure 4c,d just before and after GN coating, respectively, and this type of electrode has diameter of 24 mm and a thickness of 1 mm. ECG signals were measured after amplifying, filtering, and sampling processes using a data acquisition platform which is also shown in Figure 4e.

**Figure 5:**
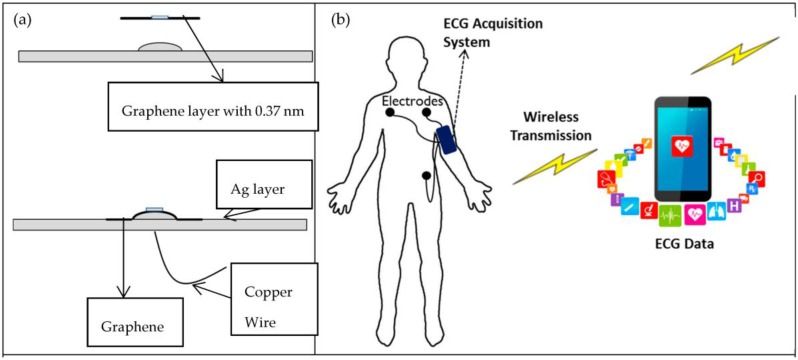
The graphene-electrode mechanism: (**a**) The mechanism of graphene-coated electrode; (**b**) the schematic illustration of the developed system while ear-lead electrocardiogram (ECG) was tested on the subject, including a wireless ECG data acquisition system.

The mechanism of graphene-based electrode is shown in Figure 5a as a separate graphene layer and Ag layer before and after the coating process. Graphene layer is designed for the purpose of the skin–electrode contact area to get the utmost ECG signal even from behind the ear location. Graphene was coated onto the dry type of Ag layer electrode. The use of graphene on dry ECG electrodes has several advantages: technically less complex (without having gel); physically attached to non-hair regions, hence more suitable for long term use; and user friendly, as there is no need to remove the top garment. On the other hand, the conventional Ag/AgCl electrode for ECG monitoring works with electrolyte gel which works as a conductive layer between the skin and electrode to lower skin–electrode contact impedance and stablise reception of the signal. However, the electrolyte gel is undesirable for long-term ECG monitoring due to its causing skin-related problems such as dermatitis. Moreover, the electrolyte gel dries out with time, which could result in degradation of signal quality and so prevents body movements for the patient at the same time. To overcome the drawbacks, the graphene-coated dry electrodes are suggested for long-term ECG monitoring with wirelessly transmission ECG data in this concept (see Figure 5b).

Furthermore, the skin–electrode contact impedance of the GN-coated electrode was measured and compared with that of the conventional Ag/AgCl pre-gelled electrode by an impedance analyser (Wayne Kerr 6500B (Wayne Kerr Electronics, West Sussex, UK)). It is known that a better ECG signal would give lower skin-to-electrode contact impedance because of high quality signal acquisition comprised of minimal noise. Results will be specified later, demonstrating the attractive performance of the GN-enabled electrode for measurements of the ECG and skin–electrode contact impedance.

## 3. Experimental Results and Discussion

### 3.1. Simulation of Electroplating Graphene

**Figure 6:**
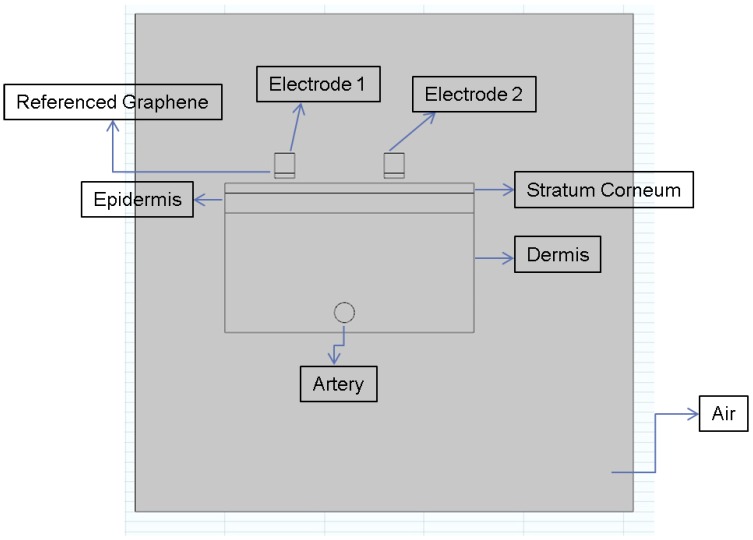
Initial skin model with two electrodes constructed in Comsol.

Since the Ag layer has already been placed on the electrode, we wanted to electroplate graphene (GN) on top of electrode to increase the surface electrical properties and to see the differences using finite element software Comsol Multiphysics. Figure 6 draws the design of skin model with each layer of the skin. Considering the fact that physiological systems are very complex in nature, here we only considered the following parameters in the construction of our skin model: (1) Stratum Corneum; (2) Epidermis; (3) Dermis; and (4) Muscle. ECG can be required by placing electrodes on a person’s skin, and coupling between electrode and skin could be described as a resistor and a capacitor in parallel [26]. The skin–electrode contact impedance is dependent on electrical conductivity or resistivity of each layer. Here, we assigned the electrical conductivity values of Stratum Corneum, Epidermis, Dermis, and Artery (Blood) as 0.0005 S/m, 0.95 S/m, 0.2 S/m, and 0.8 S/m (see in Table 1), respectively, in simulation of the mode; these values were obtained from [27]. As can be seen from the figure, there is a bottom layer of the electrode called referenced graphene. We filled in that layer with Cu first, and then graphene in order to compare the electrical characteristics. Again, simulations were performed for two types of electrodes: conventional Ag/AgCl and graphene-coated Ag/AgCl. The values of electrical conductivity were applied as 6 × 10^7^ S/m and 1 × 10^9^ S/m for Cu and GN, respectively.

**Figure 7:**
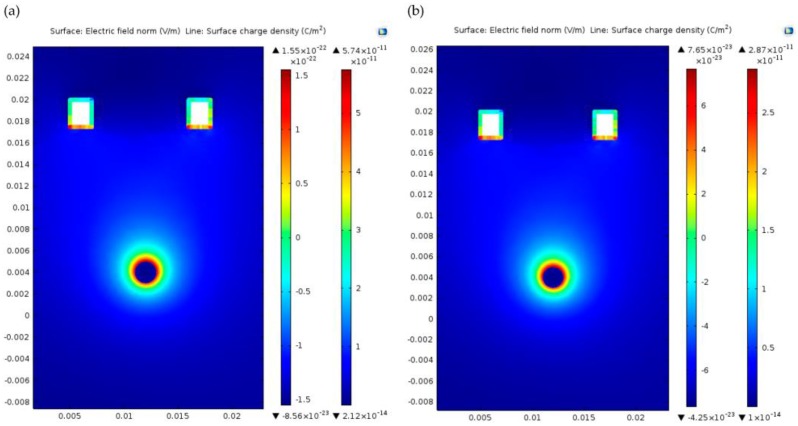
Comparison the electric field effect and surface charge density of the electrodes using GN-based (**a**) and conventional Ag/AgCl based electrode (**b**).

After GN electroplating, the concentration distribution of the electromagnetic field was depicted in Figure 7. The electrical field effect showed the electrical distribution of an artery, which is drawn in Figure 7, and the capture effect of the GN-filled electrode on top of the skin layer within current density lines. It was also analysed in Comsol that the surface charge density of the GN-coated bottom layer was higher than the Ag-filled electrode, thus capturing more electrical potential from the surface (see Figure 7a,b). The electromagnetic field and distribution was also depicted by measuring surface electric displacement field. It was visible that the GN deposition at the bottom of the electrodes was capturing electrical potential due to higher current density.

### 3.2. Measurement of Skin-Electrode Contact Impedances

**Figure 8:**
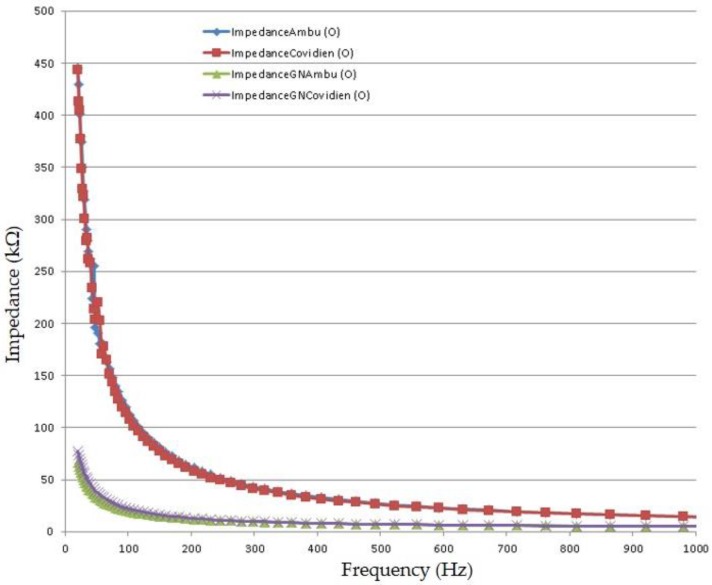
The skin–electrode contact impedance of Ambu (bigger) and Covidien (smaller) types of conventional Ag/AgCl electrode (shown as ImpedanceAmbu and ImpedanceCovidien) and graphene-coated electrodes (shown as ImpedanceGNAmbu and ImpedanceGNCovidien).

**Figure 9:**
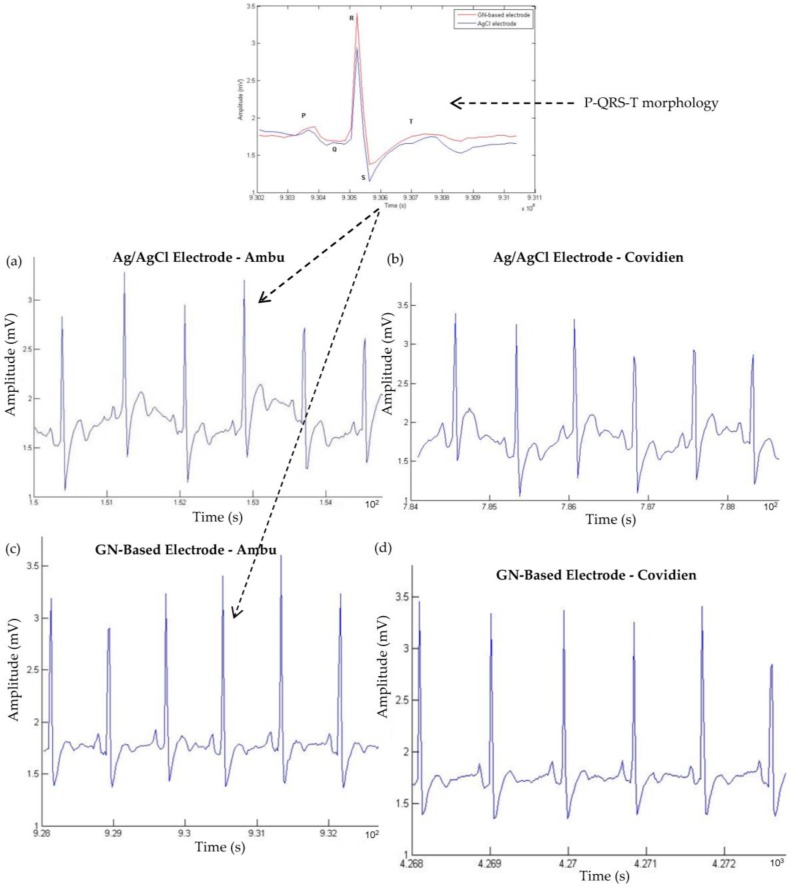
Chest-based ECG signals from the Ag/AgCl electrode and from the GN-based electrode using Ambu (larger size) and Covidien (smaller size) types of electrodes (**a**–**d**).

Skin–electrode contact impedance measurement has always been of interest due to proving the reliability of the collected biopotential. Skin conductivity varies according to variations of the conditions of either the stratum corneum (whether hair is on skin or not) or sweat proportions. In order to get a high-quality signal acquisition with minimal noise, the impedance measurement of skin–electrode should be small and stable. To characterize the impedance of the graphene-coated electrode, a measurement setup was constructed based on earlier techniques reported in the literature [4,5]. Here, we measured the electrode–skin contact impedance of three GN-coated electrodes with different sizes, and compared with that of the conventional Ag/AgCl electrode (see Figure 8). Both the conventional Ag/AgCl electrodes and the proposed GN-coated electrodes were placed adjacent to each other on a person’s forearm (between the wrist and the elbow), and both electrodes were attached so as to maintain the same distance (3 cm). Each measurement was carried out just after attaching the electrodes (in period of 45 s) and after removal of skin moisture. The impedance measurements were recorded in the frequency range of 20 Hz to 1 kHz. According to these measurements, the trend of impedances with varying frequencies was improved by graphene coating when compared to that of Baek’s [5] and Meng’s [4] results. Figure 8 shows the impedance values of conventional Ag/AgCl electrode ranges from 445.05 kΩ (at 20 Hz) to 13.82 kΩ (at 1 kHz), which are similar to results reported in the literature [4,5], and the impedance of the graphene-coated electrode varies from 65.82 kΩ (at 20 Hz) to 5.10 kΩ (at 1 kHz). The results show that the graphene-coated electrode has lower skin–electrode contact impedance compared to the conventional Ag/AgCl electrode, resulting in less noise and a higher quality ECG signal, which is shown in Figure 9. Another interesting point from Figure 8 is that the contact impedance decreases as the size of the electrode increases (as mentioned earlier, Ambu type electrode is slightly bigger than Covidien type), which results from the increased contact area between the electrode and the skin.

### 3.3. Measurements of ECG Signals

Electrocardiogram (ECG) signals were recorded using two types (Ambu and Covidien) of conventional Ag/AgCl electrodes and the newly developed GN-based electrodes at the lead I position. The typical ECG signals from each electrode are shown in Figure 9. In comparison with the ECG signals obtained with the conventional Ag/AgCl and GN-based electrodes, it is observed that there was no significant difference between the two signals, but only that the waveforms of the ECG signal from GN-coated electrode were much more stable than conventional electrodes. The P-wave, QRS-complex (QRS-complex is the combination of the Q-wave, R-wave and S-wave and represents ventricular depolarization), and the T-wave were clearly visible in both waveforms and both Ambu and Covidien types of electrodes. It is also shown that electrodes with larger sizes exhibit less noise due to a larger skin-electrode contact area. The GN-based Covidien type (smaller size) electrode provided ECG signals that are comparable to Ambu type (larger size) conventional Ag/AgCl electrode. In particular, the results of both types of GN-coated electrode demonstrated the potential use of these electrodes in clinical settings.

**Figure 10:**
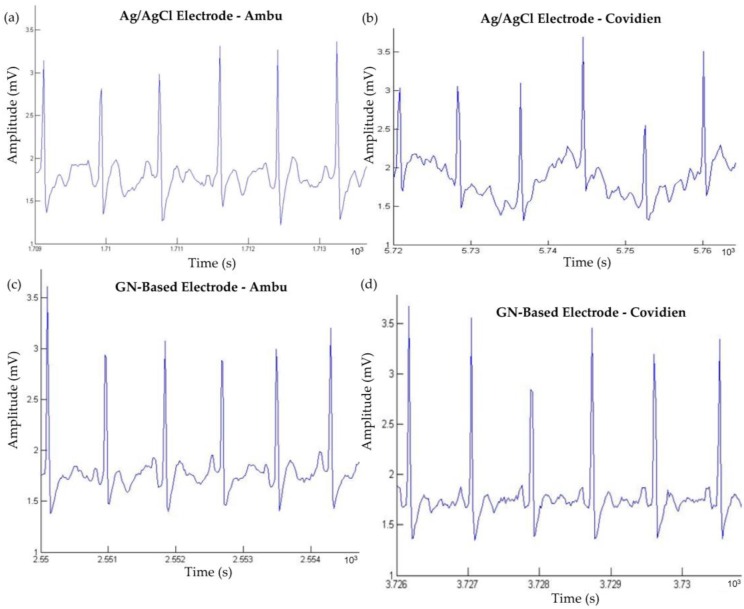
Ear-lead based ECG signals from the Ag/AgCl electrode and from the GN-based electrode using Ambu (larger size) and Covidien (smaller size) types of electrodes (**a**–**d**).

To further evaluate and compare the performance of the GN-coated and Ag/AgCl electrodes, the ear-lead electrode position was suggested to put into the system to see whether the signals are still stable, due to easy for use and simplicity for ECG setup and monitoring. Normally, it is quite difficult to measure an ECG signal near the ear due to weak surface conduction when compared to lead I position, and the body movement has a significant effect on the ECG signal. However, GN-enabled electrodes have shown a great effect on coupling ECG signals from behind-the-ear location when compared to Ag/AgCl electrodes. Here, the first electrode of a three-electrode setup was placed behind the ear; the second electrode was attached on the upper neck area, and the last electrode, called reference electrode, was placed on the left waist. Again, we also examined the effect of the size of electrodes, as Ambu and Covidien types of electrodes were used. Figure 10a,c illustrates the ECG signals of the Ag/AgCl electrode and of the GN-enabled electrode using Ambu-type electrodes located behind the ear. Likewise, we also measured the signals of Ag/AgCl electrode and of the GN-coated electrode using the Covidien type of electrode, and ECG waveforms are shown in Figure 10b,d, respectively.

As can be seen from the figure, it was discovered that there are no significant changes on the ECG signals using Ambu type of electrodes, but on the other hand, acquired ECG signals were distorted in the case of the Covidien type of electrode, and we could not identify the P- and T-waves from there (see Figure 10c). However, we were able to observe the P-wave, QRS-complex and the T-wave within Covidien type GN-based electrode as shown in Figure 10d. Moreover, due to increased contact area with the larger size of electrode (Ambu type of electrode), the quality of ECG waveforms was better than that of the Covidien type of electrode when Figure 10a–c is compared to Figure 10b–d.

Furthermore, we evaluated the performance of the electrodes, and ECG signals were analyzed to calculate signal-to-noise ratio (SNR) using the following equation [14]:

SNR = 20 log(S/(S’ − S))
(1)

where S is the filtered ECG signal with a frequency ranging from 0.5 Hz to 100 Hz, and S’ is defined as ECG signal without filtering. Before calculation, the power line interference (50 Hz) was removed from both signals. Table 2 summarizes the SNR of eight different ECG results, which are represented in Figure 9 and Figure 10, between a–d, respectively.

**Figure 11:**
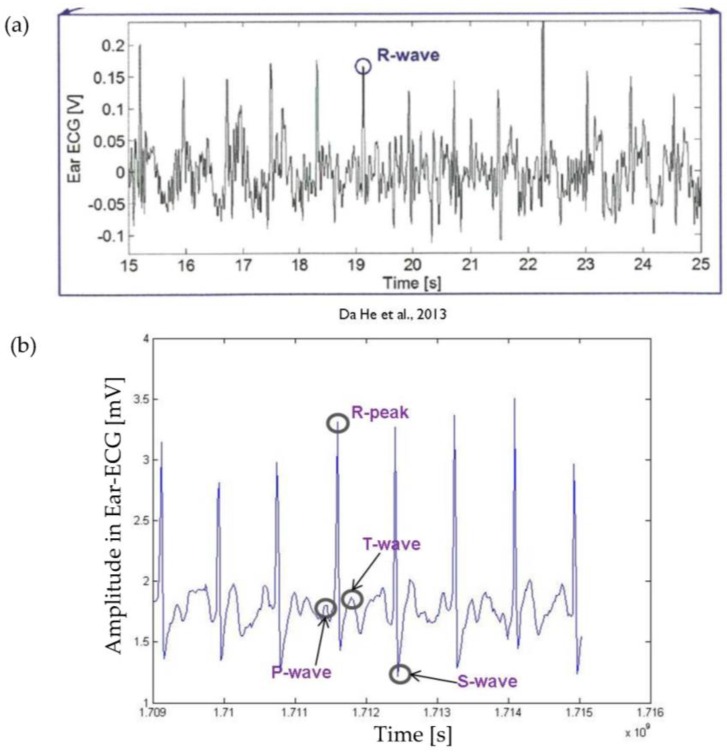
Comparison of ECG signals recorded by different electrodes. (**a**) ECG signals recorded by Ag/AgCl electrode in Da He’s report using ear-lead position (only R-peaks are visible); (**b**) ECG signals recorded by GN-based electrode using ear-lead position (all P-QRS-T morphology is identified).

Results clearly revealed that the proposed GN-based electrode within both Ambu and Covidien type electrodes provide better signal quality and performance than traditional Ag/AgCl electrodes. Another point from the results is that GN-based electrode presents a comparable SNR and ECG signal even from behind the ear, which is a quite useful location for the patients in wearable healthcare. These results also pointed out that SNRs agreed with the results taken from ECG signals (Figure 9 and Figure 10), skin–electrode contact impedance (Figure 8), simulation in Comsol (Figure 7), and the electrical conductivity values to realise the effect of GN. Meanwhile, we have also compared acquired ear-lead ECG signals by the proposed GN-based electrode with recently published work by Da He [28] who reported ECG signals obtained by Ag/AgCl gel electrodes using a behind-the-ear device. In the results reported, only R-waves appeared clearly including various noisy data, however, our results clearly identify P-waves, QRS-complex, and T-waves with less noise (see in Figure 11). The critical point here is that Da He used two active electrodes because of the limited skin area near the ear, thus DRL (reference) electrode was omitted in his experiments.

**Figure 12:**
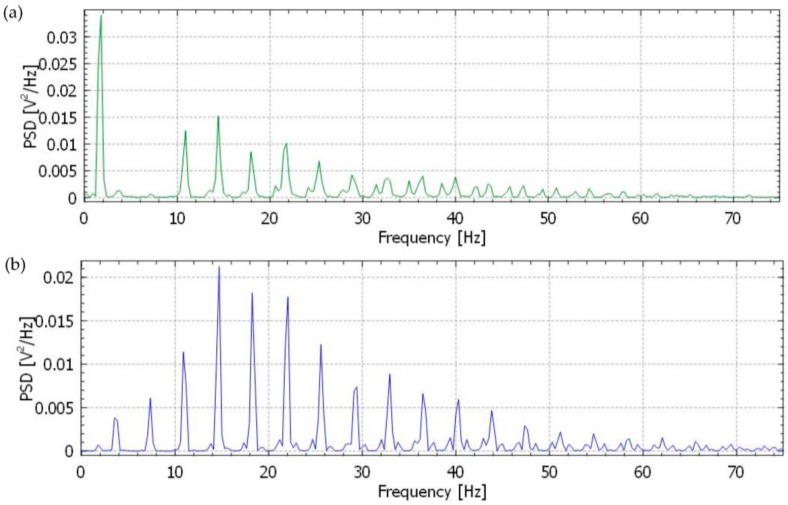
Comparison of the frequency response of filtered ECG signals from the Ag/AgCl and GN-based electrode up to 75 Hz. (**a**) Power spectral density (PSD) of ECG recordings (from Figure 8a) for the Ag/AgCl electrode; (**b**) PSD of ECG recordings (from Figure 8c) for GN-based electrode.

In addition, we also evaluated and compared the performance of the GN-based and Ag/AgCl electrodes by giving a power spectrum of the recorded ECG signals using the built-in KST (KST is a data plotting and analysis program) function [29] with a Hamming window. Figure 12a,b show the power spectral density (PSD) of the ECG signals from the both Ag/AgCl and GN-based electrodes, respectively. It is clearly seen that frequency response curves for the GN-based electrodes illustrate a better power spectrum than Ag/AgCl electrodes where the critical P-QRS-T morphology of the ECG in the 0–40 Hz frequency range is accurately captured. Analysis of the PSD also confirms that the low frequency fluctuations occur for the Ag/AgCl electrodes in the 2–5 Hz range, however the noise signal is observed after 40 Hz frequency range for the GN-based electrode. This noise signal is acceptable and caused by very low fluctuations during the ECG recording. The trend of PSD with varying frequencies was similar to that of Yapici’s PSD results [30]. According to their results, the PSD of ECG signals showed that the noise signal was minimized using Welch periodogram filtering method which is built into Matlab. Yapici et al. [30] investigated the effect of GN for fabricating a textile electrode in an ECG recording system and they analysed the characterization of electrode frequency response for further evaluation.

To investigate the stability and reliability of the GN-based electrode in long-term monitoring, we continued the experiment using our GN-based electrode on 10 different subjects for 2 months. Although same GN-based electrodes were used, we were able to clearly observe a typical ECG waveform including P-waves, QRS-complex and T-waves. Each time, the electrodes were cleaned with acetone and prepared to be tested with the next subject. After experiments with testing GN-based electrodes on 10 different subjects, we measured the resistance of the electrode to examine if such residues or microcracks affect the electrical properties of the electrode. The electrical resistances of the three electrodes were measured, and they were 0.076 ± 0.019, 0.064 ± 0.024, and 0.089 ± 0.129 Ω, respectively. Even though a small increase of resistance was discovered, the electrode still had good electrical conductivity with 416 S/m after 2 months of experiments. These experimental results showed that the GN-enabled electrode is wearable for more than 2 months and that this electrode can be used on different patients for use in long-term ECG monitoring.

## 4. Conclusions

In this paper, a novel graphene (GN)-coated ECG electrode was developed and its performance was tested in terms of quality-of-signal and durability. The electrodes were obtained by CVD grown on Cu method and the structures were transferred through Ag substrates. The experimental results clearly showed improved performance with graphene-coated electrodes. The signal-to-noise ratio has improved by 8%, significantly due to GN coating; the shape of the signal has been much better with GN-coated electrodes than that of electrodes without GN coating. Thus, ECG signal had less low-frequency fluctuation and high-frequency noise with GN-coated versus uncoated. It was also found that quality of GN-coated electrodes was not significantly degraded even after multiple uses (10 times).

The characteristics of graphene coating on the conventional electrode was also investigated experimentally using SEM images, Raman spectroscopy, and impedance measurements. The measurements/observations from these experiments have evidently showed the improvement due to graphene coating. The SEM images show the smoother surface of GN coating, which would increase skin-to-electrode contact. The Raman spectroscopy measurement confirms that there is no presence of the 2D-band before GN coating process, however, Raman spectrum consists of a pronounced 2D-peak of comparable intensity to the G-peak and a pronounced reduction in the full width at half maximum (FWHM) of the D- and G-bands (see in Figure 3a,b). As mentioned earlier, these Raman spectroscopy measurements fit with single-layer GN deposition onto the substrate, evidencing separation of itself from the case of graphite or multiple-layer GN deposition. Impedance measurements of the ECG electrodes have shown reduced impedance due to the GN coating compared to that of electrodes without coating, which would increase the sensitivity of the electrode.

A finite element modelling (FEM) of skin–electrode was also developed to understand the electrical activities of skin–electrode contact. The simulation results also suggest that the GN coating has improved the current density and electric field in the region of interest, where electrode connects to the skin. These results hold promise for further development of the new nanomaterial-enabled dry electrodes for electrophysiological sensing in wearable technologies. Therefore, the GN-based electrode reflects potential application in not only cardiac activity ECG monitoring systems, but also muscular (EMG) and neural activity (EEG).

## Figures and Tables

**Table 1:** Electrical conductivity and relative permittivity values of each layer in Comsol modelling.

Layers of Modelling	Electrical Conductivity (S/m)	Relative Permittivity
Graphene (GN)	1.18 × 10^9^	11.5
Ag-Silver	61.6 × 10^6^	3.4
Stratum Corneum	0.0005	1
Epidermis	0.95	1
Dermis	0.2	1
Artery	0.8	1

**Table 2:** Signal-to-noise ratios (SNRs) of electrocardiography (ECG) signals obtained with different electrodes and placements.

Electrode Placement	Ambu Type Electrode		Covidien Type Electrode	
	GN-based	Ag/AgCl	GN-based	Ag/AgCl
Chest-lead	27.03	25.21	25.32	21.23
Ear-lead	22.96	17.27	21.74	15.34

## Supplementary Materials

The following are available online at www.mdpi.com/2079-4991/6/9/156/s1. Figure S1: Raman analysis of bare Ag/AgCl electrode for detecting 2D band in 10 measurements, Figure S2: Raman analysis of Graphene-coated electrode for detecting 2D band in 10 measurements. Although weak peaks occurred during tests, 7/10 peaks presented in 2D band appearances.
