# Supplementary material for: Graphene-Enabled Electrodes for Electrocardiogram Monitoring
Source: Nanomaterials (Basel). 2016 Aug 23;6(9):156. doi: 10.3390/nano6090156 (PMC5224636; doi:10.3390/nano6090156)
Supplement: Supplementary file 1 [file nanomaterials-06-00156-s001.pdf]

Supplementary Materials: Graphene-Enabled Electrodes for Electrocardiogram Monitoring

Numan Celik, Nadarajah Manivannan, Andrew Strudwick and Wamadeva Balachandran

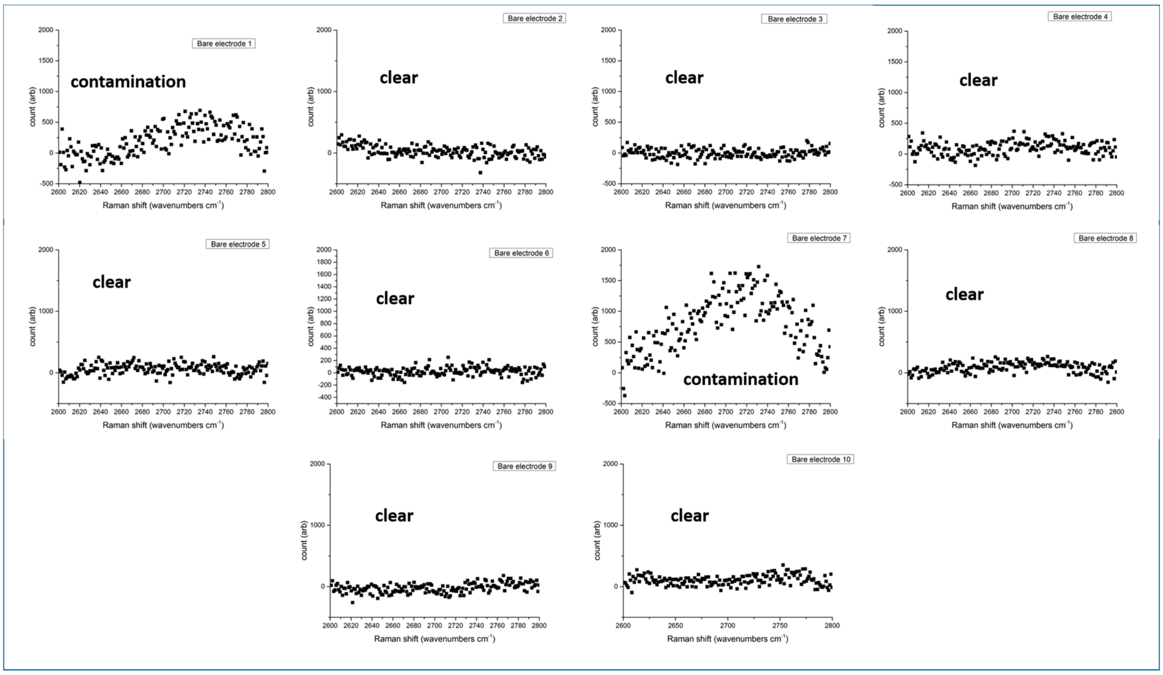

Figure S1. Raman analysis of bare Ag/AgCl electrode for detecting 2D band in 10 measurements.

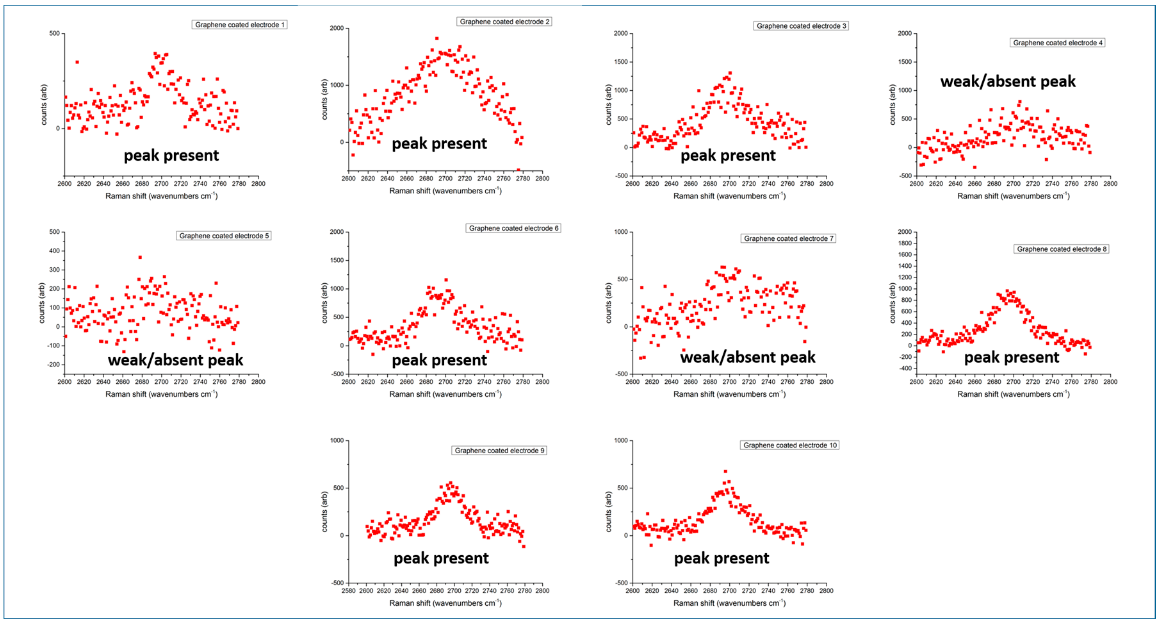

Figure S2. Raman analysis of Graphene-coated electrode for detecting 2D band in 10 measurements. Although weak peaks occurred during tests, 7/10 peaks presented in 2D band appearances.
